# Artery compliance in patients with rheumatoid arthritis: results from a case-control study

**DOI:** 10.1007/s10067-017-3899-8

**Published:** 2017-11-13

**Authors:** Lei Wang, Wenfeng Tan, Fang Wang, Youxuan Shen, Huanping Mei, Yanyan Wang, Yao Ke, Lei Gu, Qiang Wang, Miaojia Zhang

**Affiliations:** 1 Department of Rheumatology, The First Affiliated Hospital of Nanjing Medical University, Jiangsu, China; 2 Department of Cardiology, The First Affiliated Hospital of Nanjing Medical University, Jiangsu, China

**Keywords:** Arterial compliance, Atherosclerosis, Rheumatoid arthritis, Risk factors

## Abstract

Atherosclerosis is one of the most common complications of rheumatoid arthritis (RA). The objective of this study is to evaluate differences in large artery compliance (C1) and small artery compliance (C2) between RA and controls and evaluating factors associated with reduced compliance in the RA population. The profiling of large and small arterial compliance was analyzed in 185 RA patients and 88 healthy controls using Cardiovascular Profiling Instrument. The correlations of arterial compliance and the relevant clinical data were determined in these subjects. Then correlation analysis and regression analysis were performed to find whether rheumatoid arthritis patients have more risk factors than healthy controls in artery compliance and to explore the possible element involved in RA patients including traditional cardiovascular risk factors, RA disease-related factors, and the therapy. Compared with healthy controls, levels of C1 and C2 were significantly decreased in RA patients. Having adjusted the traditional risk factors associated with atherosclerosis, C1 and C2 decline was still a significant indicator in RA patients [odds ratio = 7.411(95%CI 3.275, 16.771) and 10.184(95%CI 4.546, 22.817)]. Using multi-factor regression analysis to adjust traditional risk factors for arterial compliance, we found that the levels of ESR was correlated with the abnormal large artery compliance [odds ratio = 1.021(95%CI 1.007, 1.035)]. The HAQ values and the current usage of leflunomide were correlated with the abnormal small artery compliance in RA patients [odds ratio = 1.161(95%CI 1.046, 1.289) and 6.170(95%CI 1.510, 25.215)]. The values of C1 and C2 are indicators of artery compliance in RA patients. ESR, HAQ values, and the usage of leflunomide might be possible risk factors of artery compliance. The evaluation of artery compliance could be an easy and reliable test that could help us to screen and predict cardiovascular disorders in RA patients.

**Electronic supplementary material:**

The online version of this article (10.1007/s10067-017-3899-8) contains supplementary material, which is available to authorized users.

## Background

Rheumatoid arthritis (RA) is a common autoimmune disease involving multiple systems [1]. Atherosclerosis-induced cardiovascular disease (CVD) is one of the most common complications in patients with RA [2]. Compared with normal group, RA patients have significantly increased risk of atherosclerosis, earlier time of onset, higher mortality, and earlier time of death [3, 4].

According to previous studies, increases in traditional risk factors of atherosclerosis and inflammation are the two most important causes of increased atherosclerosis risk in RA patients [5, 6]. In addition, production of auto-antibodies and application of arthritis drugs can also increase the risk of atherosclerosis in RA patients [7, 8]. Therefore, early detection of subclinical atherosclerosis in RA patients by simple, fast, noninvasive, low-cost method is of positive significance to the improvement of their prognosis.

So far, there have been many means of assessing atherosclerosis in clinical settings, such as Doppler ultrasound measurement of patients’ carotid intima-media thickness and detection of presence/absence of arterial plaque formation [9], and coronary dual-source CT or coronary angiography detection of presence/absence of atherosclerosis and degree of coronary artery stenosis [10, 11]. Although the above approaches are highly sensitive, they have cumbersome procedures, which can only assess the already-formed atherosclerotic vessels as well, without having the predictive role in subclinical atherosclerosis.

Large artery compliance (C1), also known as the capacitive compliance, refers to the ratio of diastolic volume reduction to pressure decrease, while small artery compliance (C2), also known as the oscillatory compliance, is the ratio of oscillatory changes in diastolic volume to pressure [12]. The two reflect the stiffness and elasticity of large arteries and small arteries, respectively, which are reliable methods for early detection of vascular diseases [13]. The smaller the C1 and C2, the compliance of large and small arteries is poorer and their stiffness is higher. C1 and C2 detection results have good repeatability, with results of the same subject detected at 1–4 weeks intervals having very close means [14, 15]. Easily and noninvasively detectable, C1 and C2 are thus sensitive indices reflecting early changes of atherosclerosis [16].

This study assesses the large and small artery compliance of subjects by detecting the C1 and C2 levels of RA patients and healthy controls underwent routine physical examination at the physical examination center; meanwhile, the relationship of clinical and treatment conditions with arterial compliance in RA patients is analyzed, with the aims of evaluating differences in arterial compliance between RA and controls and evaluating factors associated with reduced compliance in the RA population.

## Methods

### Clinical data collection

Totally, 273 subjects were enrolled in this study, including 185 RA outpatients treated at the Department of Rheumatology, the First Affiliated Hospital of Nanjing Medical University, and 88 volunteers received routine physical examination at the same hospital’s physical examination center. Exclusion criteria were as follows: patients with severe infection, perioperative patients, patients with severe liver and kidney dysfunctions, cancer patients, menstrual patients, or patients who underwent coronary stent implantation or bypass graft. This study was approved by the Ethics Committee of the First Affiliated Hospital of Nanjing Medical University. Furthermore, all subjects signed an informed consent.

Clinical data of all subjects were collected in detail, including name, contact information, and traditional atherosclerosis risk factors (such as age, gender, body mass index, history of smoking, systolic blood pressure, diastolic blood pressure, fasting blood glucose, triglycerides, total cholesterol, low density lipoprotein, and high density lipoprotein), as well as RA-related factors (including course of disease, tender joint count, swollen joint count, morning stiffness, erythrocyte sedimentation rate, C-reactive protein, visual analogue scale scoring, patient global assessment scoring, evaluator global assessment scoring, simplified disease activity index scoring, clinical disease activity index scoring, disease activity score 28 joint count, health assessment questionnaire scoring, rheumatoid factor and anti-CCP antibody positive/ negative, drug in use or not, including non-steroidal anti-inflammatory drugs, glucocorticoids, disease modifying anti-rheumatic drugs, methotrexate, leflunomide and hydroxychloroquine).

### Determination of arterial compliance and measurement of C1 and C2

Radial arterial pulse waveforms were recorded with radial artery diastolic pulse waveform analyzer (CVProfilor DO-2020, HDI, USA). According to the operation instruction, patients were connected to the instrument properly. The instrument’s software system automatically identified and collected waveform data and calculated, displayed the pressure waveform and arterial elastic function data, including systolic blood pressure (SBP), diastolic blood pressure (DBP), mean arterial pressure (MAP), pulse pressure (PP), pulse rate (PR), larger artery compliance index C1 (ml/mmHg*10), and small artery compliance index C2 (ml/mmHg*100).

According to the operating instructions of the instrument and relevant previous literature, a subject was considered to have abnormal large arterial compliance when his/her mean C1 of three test results was less than 10 ml/mmHg*10 and considered to have abnormal small arterial compliance when the mean C2 was less than 4 ml/mmHg*100. The test was performed three times for each subject.

### Statistics

Data were entered and self-tested using Microsoft Office Excel 2007 and statistically analyzed using IBM SPSS Statistics 19.0. Before the analysis, hypothesis testing was performed using normal probability plots to observe whether the detected values followed normal distribution. Normal distribution measurement data were expressed as mean ± standard deviation, whereas the skewed distribution measurement data were expressed as median. The two sets of measurement data were compared by Student’s *t* test, while comparison of categorical data was done by Pearson’s chi-square test. *p* < 0.05 was considered statistically significant, and all statistical tests were two-tailed probability tests. We performed correlation analysis between the value of C1, C2, and RA patients or healthy controls, items of traditional atherosclerosis risk factors and RA-related factors, respectively. Also, we put RA patients or not and traditional atherosclerosis risk factors into the regression analysis of model 1, and all items of traditional atherosclerosis risk factors and RA-related factors were putted into the regression analysis of model 2. Correlation analysis and regression analysis were performed using SPSS 19.0.

## Results

### Analysis of basic information, clinical data, and arterial compliance

RA group and healthy control group did not show significant differences (*p* > 0.05) in gender ratio, age, BMI, waist circumference, SBP, DBP, MAP, PP, fasting blood glucose (FBG), triglycerides, high density lipoprotein (HDL), history of smoking, history of hypertension, history of diabetes or history of hyperlipemia. Compared with the healthy control group, bodyheight, weight, total cholesterol (TC), and low density lipoprotein (LDL) were significantly lower in the RA group (*p* < 0.05), while PR was significantly higher (*p* < 0.05). In addition, compared with the healthy control group, C1 and C2 significantly reduced in the RA group, while proportions of abnormal large and small artery compliance increased significantly (*p* < 0.001) (Table 1).

**Table 1:** Basic information and laboratory index of subjects

	RA	HC	*p* value
Case number	185	88	
Gender	153; 82.7%	66; 75.0%	0.146
Age (years)	51.66 ± 11.77	49.67 ± 7.18	0.086
Height (centimeter)	161.09 ± 6.69	164.18 ± 6.96	0.001
Weight (kilogram)	61.28 ± 9.726	65.19 ± 11.70	0.004
BMI	23.59 ± 3.33	24.09 ± 3.30	0.244
Waist (centimeter)	85.82 ± 10.10	85.08 ± 9.96	0.569
SBP (mmHg)	128.70 ± 17.24	125.20 ± 16.91	0.117
DBP (mmHg)	74.84 ± 10.29	73.53 ± 10.63	0.334
MAP (mmHg)	92.22 ± 17.37	92.17 ± 13.53	0.981
PP (mmHg)	53.28 ± 12.76	51.82 ± 8.95	0.275
PR (per minute)	77.84 ± 15.57	73.58 ± 10.68	0.021
FBG (mmol/L)	5.37 ± 0.82	5.20 ± 0.51	0.056
TG (mmol/L)	1.20 ± 0.69	1.38 ± 0.96	0.093
TC (mmol/L)	4.94 ± 1.06	5.37 ± 1.01	0.003
LDL (mmol/L)	3.12 ± 0.80	3.37 ± 0.74	0.017
HDL (mmol/L)	1.40 ± 0.34	1.43 ± 0.33	0.422
History of smoking	21 (11.4%)	5 (5.7%)	0.186
History of hypertension	40 (21.6%)	23 (26.1%)	0.444
History of diabetes	5 (2.7%)	2 (2.3%)	1.000
History of hyperlipemia	5 (2.7%)	1 (1.1%)	0.668
C1 (ml/mmHg*10)	10.23 ± 4.29	12.38 ± 3.24	0.000
C2 (ml/mmHg*100)	3.24 ± 1.88	5.23 ± 2.31	0.000
Large artery compliance abnormalities	99 (53.5%)	12 (13.6%)	0.000
Small artery compliance abnormalities	141 (76.2%)	28 (31.8%)	0.000

Student’s *t* test and Pearson’s chi-square test were used to analysis. Data are mean + SD or *N*(%). Gender are *N*(%) of female

*RA rheumatoid arthritis, *HC* healthy controls, *BMI* Body Mass Index, *SBP* systolic blood pressure, *DBP* diastolic blood pressure, *MAP* mean blood pressure, *PP* pulse pressure, *PR* pulse rate, *FBG* fasting blood glucose, *TG* triglycerides, *TC* total cholesterol, *LDL* low density lipoprotein, *HDL* high density lipoprotein

Characteristics of RA cohort were listed on Table 2. Duration of RA varies from initial attack to over 40 years, with a median of 4 years, which covers a wide range. Disease activity in RA patients was assessed using DAS28 scoring system, which revealed that 63 patients were experiencing severe disease activity, 70 patients were experiencing moderate disease activity, and 39 patients were experiencing mild disease activity or remission. So in this cohort, there are relatively many patients with active disease. Among them, 116 used disease-modifying anti-rheumatic drugs, while 69 were baseline patients who did not receive regular treatment (Table 2).

**Table 2:** Characteristics of RA cohort

Disease duration (month), median (IQR)	48 (16.5–120)
RF or anti-CCP positive, n(%)	119 (64.32%)
ESR (mm/H), mean + SE	33.20 ± 2.30
CRP (mg/L), mean + SE	19.48 ± 2.44
Tender Joint Count, mean + SE	6.55 ± 0.46
Swollen joint count, mean + SE	3.66 ± 0.33
DAS28, mean + SE	4.06 ± 0.11
SDAI, mean + SE	21.04 ± 1.21
CDAI, mean + SE	19.48 ± 1.04
HAQ, median (IQR)	5 (1–13)
Treatment—NSAIDs in use, *n*(%)	77 (41.62%)
Glucocorticoids in use, *n*(%)	56 (30.27%)
DMARDs in use, *n*(%)	116 (62.70%)
-- MTX in use, *n*(%)	61 (52.59%)
LEF in use, *n*(%)	53 (45.69%)
HCQ in use, *n*(%)	56 (48.28%)

Data are median, mean ± SE or *N*(%)

*RA* rheumatoid arthritis, *RF* rheumatoid factor, *anti-CCP* anti-cyclic citrullinated peptide antibody, *ESR* erythrocyte sedimentation rate, *CRP* C-reactive protein, *DAS28* disease activity score 28 joint count, *SDAI* simplified disease activity index scoring, *CDAI* clinical disease activity index scoring, *HAQ* health assessment questionnaire scoring, *NSAIDs* non-steroidal anti-inflammatory drugs, *DMARDs* disease modifying anti-rheumatic drugs, *MTX* methotrexate, *LEF* leflunomide, *HCQ* hydroxychloroquine

**Fig. 1:**
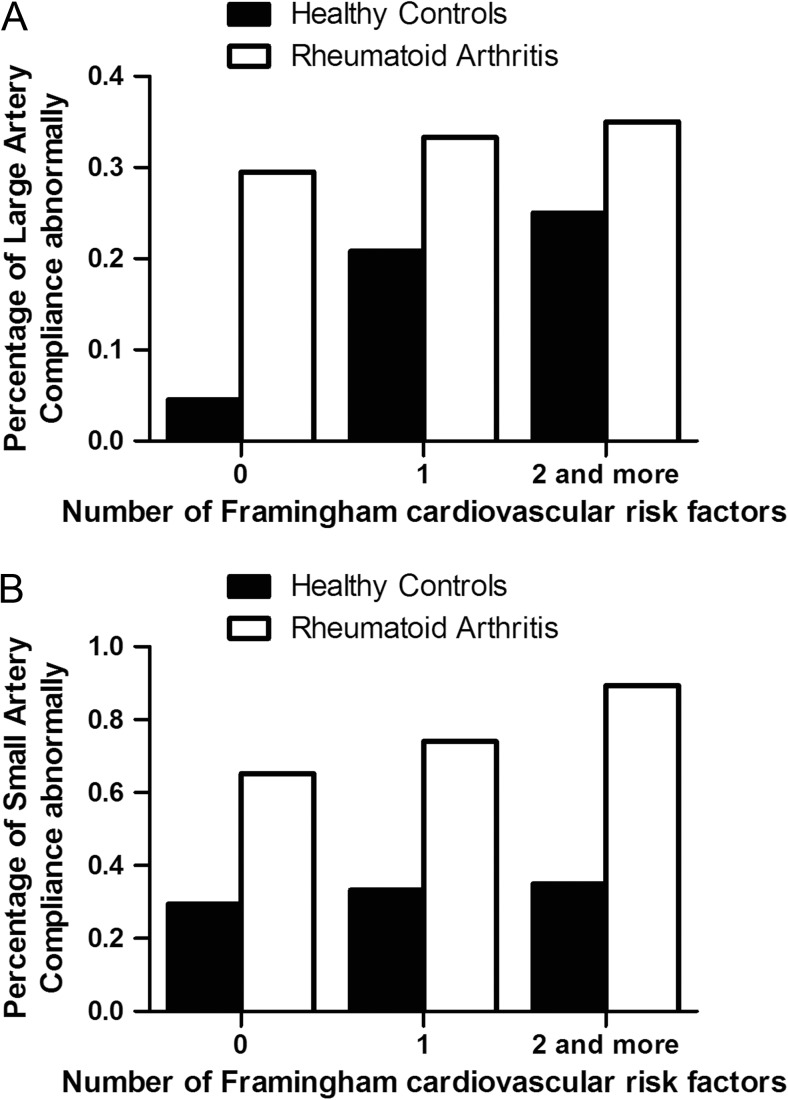
Ratio of arterial compliance abnormalities in different numbers of Framingham cardiovascular risk score. **a**–**b** The percentage of large and small artery compliance abnormally are determined by Cardiovascular Profiling Instrument between the subgroups with different number of Framingham cardiovascular risk factors

Framingham cardiovascular risk score is a common-used and credible way to assess the possibility of cardiovascular incidence occurred in the future. Age, total cholesterol, high density lipoprotein, systolic blood pressure, and smoking are contained in Framingham cardiovascular risk score. We calculated the number of risk factors in each subjects, and we found that with the increasing traditional cardiovascular risk factors, subjects’ proportions of large and small arterial compliance abnormalities increased. Moreover, in the same state of traditional risk factors, the proportions of large and small arterial compliance abnormalities were both higher for RA patients than the healthy controls (Fig. 1).

### Univariate analysis of factors influencing C1 and C2

With respect to univariate analysis among RA patients and healthy controls, age, SBP, DBP, and FBG were negatively correlated with C1 (*p* < 0.05); besides, C1 was significantly lower for RA patients than healthy controls, and for women than men (*p* < 0.05). Age, SBP, and DBP were negatively correlated with C2 (*p* < 0.05); besides, C2 was significantly lower for RA patients than healthy controls, for women than men, for men with a history of smoking than those without (*p* < 0.05).

**Fig. 2:**
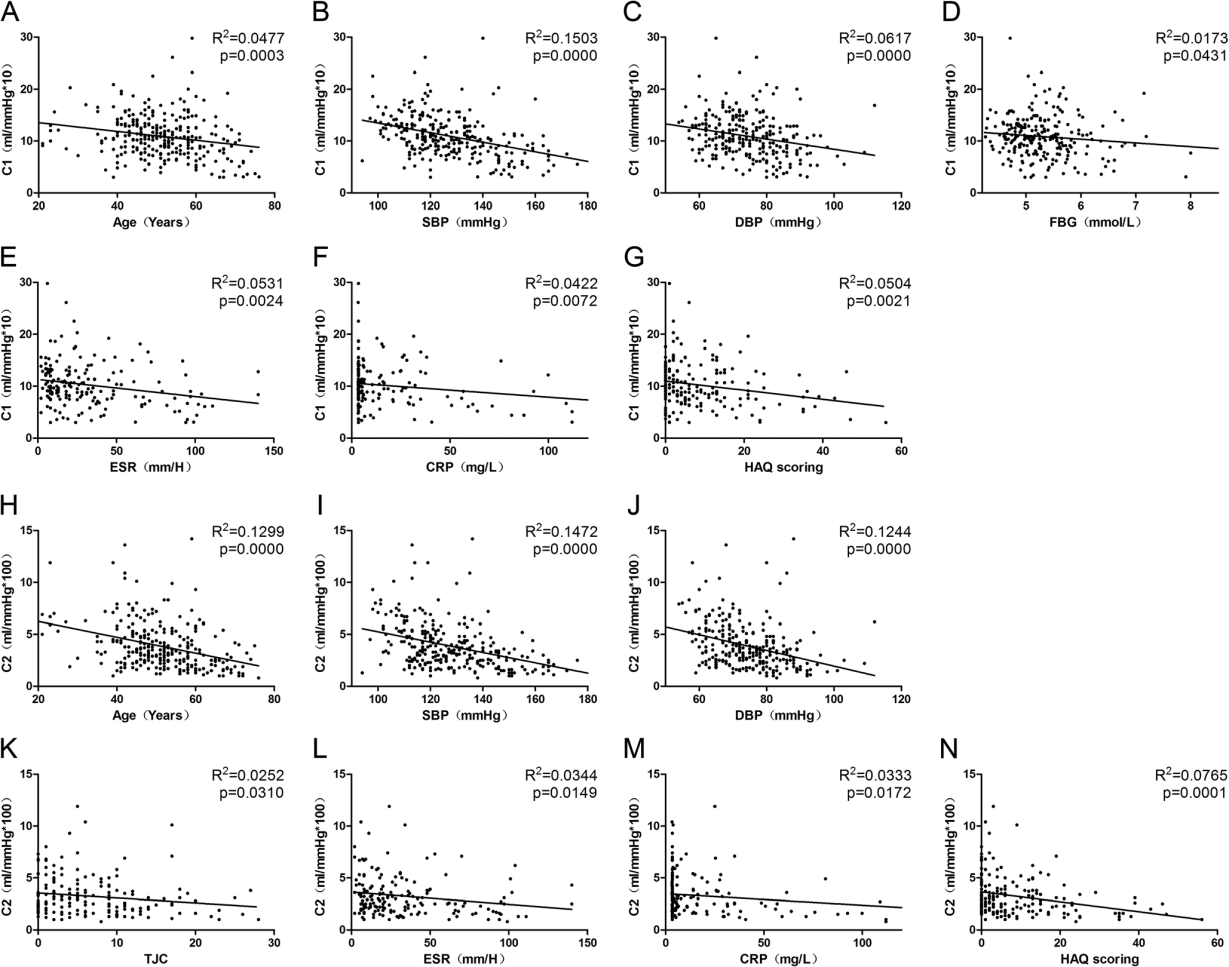
Correlation between clinical data and C1 and C2, respectively. **a**–**n** The levels of C1 and C2 are negatively correlated with clinical data

**Fig. 3:**
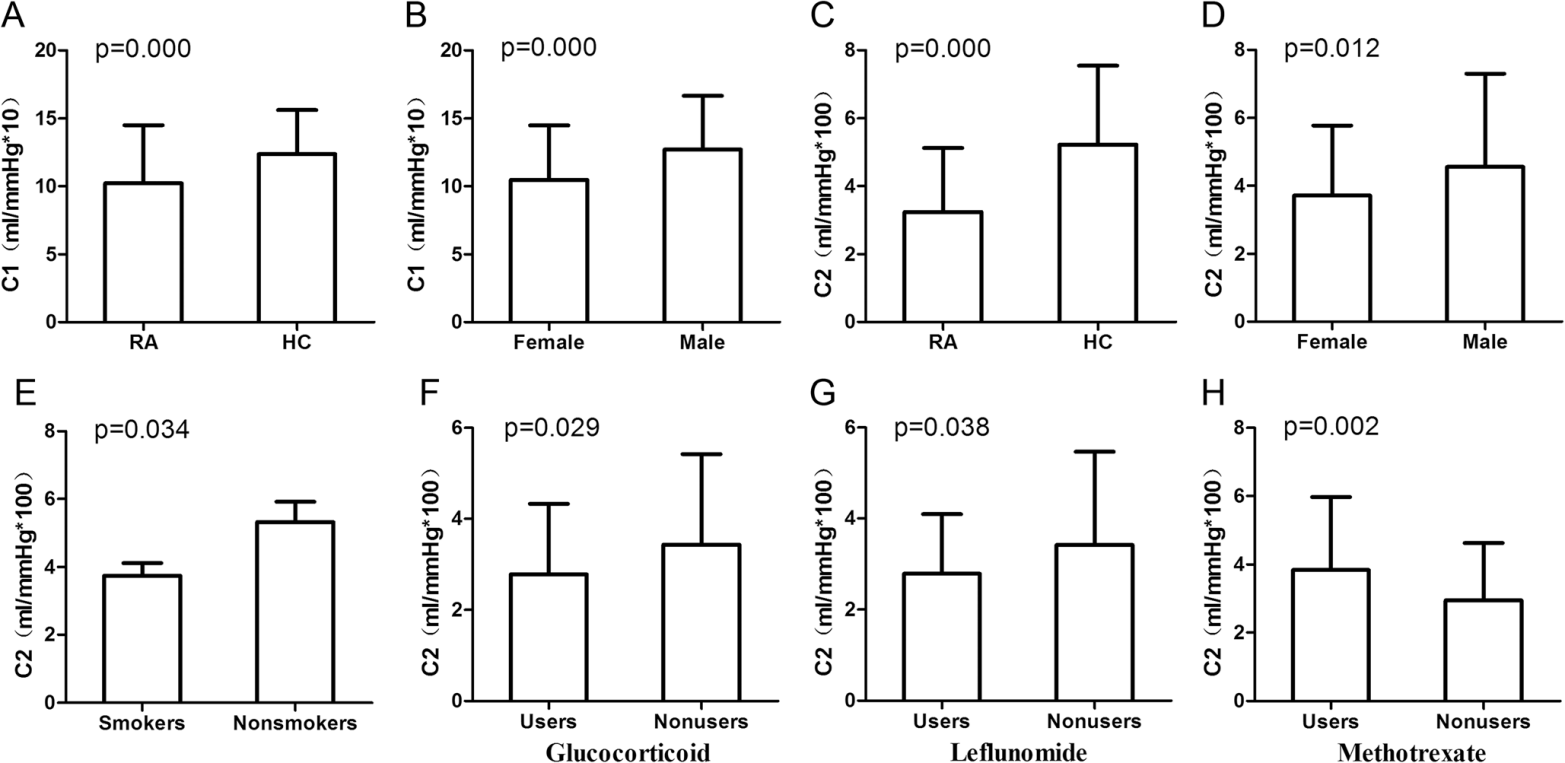
The value of C1 and C2 between different subgroups. **a**–**h**. The levels of C1 and C2 are presented between different subgroups

And in the univariate analysis of RA patients, we found ESR, CRP, and HAQ score were negatively correlated with C1 and C2 (*p* < 0.05); besides, TJC was also negatively correlated with C2 (*p* < 0.05). C2 was significantly lower for glucocorticoid users than nonusers, and for leflunomide users than nonusers (*p* < 0.05). Meanwhile, methotrexate users had significantly higher C2 than nonusers (*p* < 0.05) (Figs. 2 and 3).

### Multivariate analysis of influences of traditional risk factors and RA on arterial compliance

In the multivariate regression analysis after adjusting traditional risk factors in the Framingham cardiovascular risk score, C1 and C2 decline was still a significant indicator in RA patients, where OR values were 7.411 and 10.184, respectively (*p* < 0.05) (Table 3).

**Table 3:** Multivariate analysis of artery compliance influenced by traditional risk factors, rheumatoid arthritis and RA-related factors

	Unadjusted		Adjusted	
	OR (95%CI)	*p* value	OR (95%CI)	*p* value
Large arterial compliance				
Model 1				
Female	2.272 (1.169–4.415)	0.016	4.185 (1.506–11.627)	0.006
BMI	0.982 (0.912–1.057)	0.623	0.875 (0.784–0.977)	0.018
SBP	1.060 (1.041–1.079)	0.000	1.124 (1.078–1.172)	0.000
HDL	0.567 (0.251–1.283)	0.173	0.309 (0.109–0.877)	0.027
RA	7.291 (3.716–14.303)	0.000	7.411 (3.275–16.771)	0.000
Model 2				
SBP	1.060 (1.041–1.079)	0.000	1.058 (1.030–1.088)	0.000
ESR	1.014 (1.004–1.026)	0.008	1.021 (1.007–1.035)	0.003
Small arterial compliance				
Model 1				
Age	1.078 (1.048–1.109)	0.000	1.085 (1.042–1.129)	0.000
Female	2.026 (1.110–3.699)	0.021	7.966 (2.777–22.850)	0.000
BMI	0.999 (0.928–1.075)	0.976	0.821 (0.720–0.937)	0.004
SBP	1.062 (1.041–1.083)	0.000	1.059 (1.009–1.112)	0.021
DBP	1.094 (1.061–1.127)	0.000	1.097 (1.021–1.178)	0.012
RA	6.867 (3.915–12.045)	0.000	10.184 (4.546–22.817)	0.000
Model 2				
Age	1.078 (1.048–1.109)	0.000	1.127 (1.056–1.202)	0.000
BMI	0.999 (0.928–1.075)	0.976	0.760 (0.620–0.931)	0.008
DBP	1.094 (1.061–1.127)	0.000	1.204 (1.106–1.312)	0.000
HAQ scoring	1.068 (1.017–1.122)	0.008	1.161 (1.046–1.289)	0.005
Leflunomide	2.559 (1.060–6.178)	0.037	6.170 (1.510–25.215)	0.011

Binary logistical regression analysis was used in regression equation of both models. Rheumatoid arthritis patients or not and all items of traditional cardiovascular risk factors (including age, female, body mass index, history of smoking, systolic blood pressure, diastolic blood pressure, fasting blood glucose, triglycerides, total cholesterol, low-density lipoprotein, and high-density lipoprotein) were putted into the regression analysis of model1. All items of traditional cardiovascular risk factors and disease-related factors (including course of disease, tender joint count, swollen joint count, morning stiffness, erythrocyte sedimentation rate, C-reactive protein, visual analogue scale scoring, patient global assessment scoring, evaluator global assessment scoring, simplified disease activity index scoring, clinical disease activity index scoring, disease activity score 28 joint count, health assessment questionnaire scoring, rheumatoid factor and anti-CCP antibody positive/ negative, drug in use or not, including non-steroidal anti-inflammatory drugs, glucocorticoids, disease modifying anti-rheumatic drugs, methotrexate, leflunomide and hydroxychloroquine) were putted into the regression analysis of model2

### Multivariate analysis of influences of traditional risk factors and RA-related factors on arterial compliance

In the multivariate regression analysis after adjusting traditional risk factors in the Framingham cardiovascular risk score, ESR was the risk factor for abnormal large arterial compliance (OR value = 1.021), whereas HAQ score (OR value = 1.161) and use of leflunomide (OR value = 6.170) were the risk factors of abnormal small arterial compliance (*p* < 0.05) (Table 3).

## Discussion

By measuring the large artery compliance C1 and small artery compliance C2 of subjects using radial artery diastolic pulse waveform analyzer, this study confirms that the C1 and C2 indices are significantly lower in RA patients compared with the healthy control group. Furthermore, after removing traditional risk factors, OR values of RA against large and small artery compliance abnormalities were 7.411 and 10.184, respectively, indicating that RA patients have evident abnormal arterial compliance and significantly increased risk of atherosclerosis.

Conventional view holds that atherosclerosis is a lipid metabolism disorder involving arteries [17]. However, with the deepening of research on vascular injury mechanisms, it has been discovered that inflammation plays an important role in the occurrence and development of atherosclerosis [18, 19]. Risk of atherosclerotic cardiovascular diseases increased significantly in patients with chronic inflammation and autoimmune diseases [20, 21]. In a chronic inflammatory state, some inflammatory mediators such as tumor necrosis factor (TNF), interleukin (IL)-1, and IL-6 can cause endothelial cell dysfunction and increased protein levels of adhesion molecules and chemokines on endothelial cell surface [22–25], thus contributing to the recruitment of monocytes into the arterial wall composed of endothelial cells; meanwhile, macrophages express scavenger receptors to promote the formation of foam cells, ultimately leading to the occurrence and development of atherosclerosis [26]. In addition, increased traditional risk factors of atherosclerosis are considered to be another important cause of increased atherosclerosis risk in RA patients [27]. RA patients have significantly increased traditional risk factors of atherosclerosis such as smoking, hypertension, diabetes, dyslipidemia, obesity, and decreased activity than the general population. Therefore, EULAR recommended in 2016 that Framingham scores should be multiplied by 1.5 in the calculation of atherosclerosis risk factors for patients with RA [28].

In this study, arterial compliance of 185 RA patients and 88 healthy controls are measured with radial artery diastolic pulse waveform analyzer and analyzed by combining relevant clinical data. On the basis of matching gender and age, we found no significant difference in the degree of obesity, blood pressure, FBG level, smoking history, or chronic disease history between the two groups. However, RA patients had reduced height, weight, TC level, LDL level, and elevated PR compared with healthy controls. These differences have been published in the literatures, which, on the one hand, are considered to be associated with the poor living standard and nutritional status of RA patients and, on the other hand, are possibly associated with the occurrence of rheumatoid “cachexia” state.

In the analysis of arterial compliance between the two groups, we found that the C1 and C2 absolute values significantly reduced while the proportions of large and small arterial compliance abnormalities increased significantly in RA patients. Meanwhile, we recommended all RA patients with abnormal large and small artery compliance receive ECG and carotid B-ultrasound examinations. Some patients already presented with myocardial ischemia, carotid intima-media thickening, plaque formation, and other atherosclerotic manifestations. Besides, we will follow up other RA patients with abnormal large and small artery compliance but without presenting abnormalities on ECG or carotid B-ultrasound in the long term, in order to clarify the value of this method for predicting atherosclerosis in RA patients.

This study suggests that ESR, CRP, and HAQ score are important risk factors of abnormal arterial compliance in RA patients [29]. Therefore, patients’ disease activity should be reduced as early as possible, in order to reduce inflammatory markers of patients with active RA and improve their activity function, which help reduce their risk of atherosclerosis. In the treatment of arthritis, methotrexate is a protective factor against abnormal arterial compliance and atherosclerosis, while glucocorticoids and leflunomide are the risk factors. Thus, minimization of the dose and duration of glucocorticoids is conducive to reducing the atherosclerosis risk in RA patients. Meanwhile, the relationship between leflunomide and atherosclerosis needs further investigation.

It has been recognized that arthritis disease control and resumption of patients’ normal activity can both significantly improve the atherosclerosis risk of RA patients [30]. Powerful anti-inflammatory action of glucocorticoids can rapidly relieve joint symptoms and systemic inflammation and help reduce the risk of atherosclerosis in RA patients. But on the other hand, the hypertensive, hyperglycemic, and fat redistribution-promoting side effects of glucocorticoids may increase the atherosclerosis risk in RA patients. Some scholars have confirmed that although glucocorticoids can significantly relieve patient conditions, they can also significantly increase RA patients’ risk of atherosclerosis [31, 32]. In addition, disease-modifying anti-rheumatic drugs represented by methotrexate, leflunomide, and hydroxychloroquine significantly improved and delayed the disease progression in RA patients [33]. At present, some small-sample studies have confirmed the positive effects of methotrexate, hydroxychloroquine, and related drugs on atherosclerosis risk reduction in RA patients [30, 34]. But extensive large-sample cohort studies, randomized controlled trials, and long-term follow-up studies are still needed for confirmation of such effects.

The effect of leflunomide on atherosclerotic risk in RA patients is still unclear in part due to sample size. The association between leflunomide and artery compliance is an interesting point that we found in our research. *Im CH* et.al performed carotid B-ultrasound examination in 406 RA patients [35]. Their results showed that among 148 patients with carotid plaque formation, 101 used leflunomide, while among 258 patients without carotid plaque formation, 161 used leflunomide, exhibiting a significant difference (*p* = 0.047) between two groups. In our cohort, when analyzing the effect of leflunomide to the artery compliance, we divided 185 RA patients into two groups according to leflunomide treatment or not. We analyze the effect of leflunomide to the artery compliance only based on medical treatment. However, the impact of the interactions of drugs, doses, dosing time, and etc. were ignored. Our data only suggest a potential link between leflunomide and artery compliance rather than a causal link between them. In the future, a randomized, double-blind, placebo-controlled studies and follow-up study are needed to confirm the link between leflunomide and artery compliance.

In addition, there might be association between artery compliance and treatment of leflunomide in RA patients; more clinical studies are needed to improve it.

## Study limitation

It is not matched perfectly in some items related with atherosclerosis between RA patients and healthy controls, such as age, gender, BMI, blood pressure, level of serum lipid and blood glucose, and life style including smoking and alcohol consumption. And we just receive the cross-sectional date of the patients and healthy controls at present. So, further large-sample cohort studies and randomized clinical trials should be done to demonstrate the aforementioned findings and acquire the change of artery compliance after the therapy.

## Conclusion

The values of C1 and C2 are indicators of artery compliance in RA patients. ESR, HAQ values, and the usage of leflunomide might be possible risk factors of artery compliance. The evaluation of artery compliance could be an easy and reliable test that could help us to screen and predict cardiovascular disorders in RA patients.

## Electronic supplementary material

Supplementary Table 1 (DOCX 16 kb)
