# Supplementary material for: Artery compliance in patients with rheumatoid arthritis: results from a case-control study
Source: Clin Rheumatol. 2017 Nov 13;37(1):169–77. doi: 10.1007/s10067-017-3899-8 (PMC5754458; doi:10.1007/s10067-017-3899-8)
Supplement: Supplementary file 1 — (DOCX 16 kb) [file 10067_2017_3899_MOESM1_ESM.docx]

**Supplementary Table1. The relationship between medical treatment and abnormal artery compliance in RA patients**

|  | **Number** | | **Abnormal Large Artery Compliance** | | **Abnormal Small Artery Compliance** | |
| --- | --- | --- | --- | --- | --- | --- |
|  | **Used** | **Non-used** | **OR** | ***p* value** | **OR** | ***p* value** |
| **Glucocorticoids** | 56 | 129 | 0.665 (0.354-1.248) | 0.203 | 2.323 (1.001-5.388) | 0.046 |
| **DMARDs** | 116 | 69 | 0.750 (0.412-1.369) | 0.348 | 0.833 (0.41-1.695) | 0.614 |
| **Methotrexate** | 61 | 55 | 0.868 (0.418-1.800) | 0.703 | 0.401 (0.164-0.979) | 0.045 |
| **Leflunomide** | 53 | 63 | 2.720 (1.279-5.787) | 0.009 | 3.526 (1.365-9.108) | 0.009 |
| **Hydroxychloroquine** | 56 | 60 | 0.935 (0.451-1.938) | 0.858 | 1.753 (0.742-4.143) | 0.201 |

* RA = Rheumatoid Arthritis; DMARDs = Disease Modifying Anti-rheumatic Drugs; OR = odd ratio.

*The data of methotrexate, leflunomide and hydroxychloroquine were analysis in 116 RA patients treated with DMARDs.

*Pearson’s Chi-square test were used to analysis.
